# Impact of metabolic syndrome on the progression of coronary calcium and of coronary artery disease assessed by repeated cardiac computed tomography scans

**DOI:** 10.1186/s12933-016-0404-7

**Published:** 2016-06-28

**Authors:** Lee Kyung Kim, Ji Won Yoon, Dong-Hwa Lee, Kyoung Min Kim, Sung Hee Choi, Kyong Soo Park, Hak Chul Jang, Min-Kyung Kim, Hyo Eun Park, Su-Yeon Choi, Soo Lim

**Affiliations:** Internal Medicine, Seoul National University College of Medicine, Seoul National University Hospital, 101, Daehak-ro, Jongno-gu, Seoul, South Korea; Internal Medicine, Seoul National University Bundang Hospital, 300 Gumi-dong, Bundang-Gu, Seongnam, 463-707 South Korea; Internal Medicine, Seoul National University Hospital Healthcare System Gangnam Center, 39th FL. Gangnam Finance Center, 737 Yeoksam-dong, Gangnam-gu, Seoul, 135-984 South Korea

**Keywords:** Metabolic syndrome, Coronary artery calcium, Coronary artery disease, Longitudinal cohort study

## Abstract

**Background:**

It is not clear how severe metabolic syndrome (MS) affects the development of coronary atherosclerosis.

**Methods:**

This was an observational, retrospective cohort study with Koreans who received health check-ups voluntarily. A total of 2426 subjects had baseline and follow-up coronary artery calcium score (CACS) data. Among them, 1079 had coronary computed tomography angiography (CCTA) data. We compared baseline CACS and any progression in subjects with and without MS. A more detailed analysis was conducted for coronary artery disease (CAD), which was defined by coronary artery stenosis (≥50 %), multivessel involvement, and coronary plaques in those patients with CCTA data.

**Results:**

At baseline, subjects with MS (34.0 %, *n* = 825) had higher CACS and more significant coronary artery stenosis, multivessel involvement, and atheromatous plaques than those without MS (*P* < 0.05 for all). In the follow-up (median 1197 days), subjects with MS showed significant increases in CACS and progression of CAD compared with counterparts without MS, in parallel with the numbers of MS components. Finally, MS was a significant predictor for the progression of CACS (hazard ratio 1.32; 95 % confidence interval 1.06–1.64) and progression of coronary artery stenosis and/or development of vulnerable plaque (hazard ratio 1.47, 95 % confidence interval 1.01–2.15) after adjusting for other cardiovascular risk factors.

**Conclusions:**

Subjects with MS showed progression of CAD as assessed by CACS and CCTA over ~3 years. Therefore, more vigilant screening for coronary vascular health is needed among those with MS.

**Electronic supplementary material:**

The online version of this article (doi:10.1186/s12933-016-0404-7) contains supplementary material, which is available to authorized users.

## Background

Cardiovascular disease is the leading cause of deaths worldwide [1]. According to a report of the World Health Organization in 2009, 30 % of all global deaths were attributed to cardiovascular diseases in 2008. It is also estimated that by 2030, over 23 million people will die from cardiovascular diseases each year [2, 3]. Thus, there is a major social cost in the management of cardiovascular disease.

Under these circumstances, it is worthwhile to prescreen subjects for subclinical coronary atherosclerosis and manage risk factors to prevent its development and progression. There are several methods of screening for cardiovascular disease, such as electrocardiograms, the ankle-to-brachial blood pressure index, pulse wave velocity, carotid intima-media thickness, *N*-terminal pro-brain natriuretic peptide, the presence of carotid plaques, coronary artery calcium score (CACS), and coronary artery stenosis [4–8].

Among these, CACS is used widely as a screening method [9]. CACS reflects the presence and the extent of coronary atherosclerosis and has an independent association with cardiovascular events [10]. It has been used for individualized risk stratification and for predicting outcomes [11, 12]. The American Heart Association has suggested that CACS is a reasonable tool to assess the risk of cardiovascular disease [13]. Moreover, repeated CACS measures were found to be useful in predicting cardiovascular outcomes and in assessing the effectiveness of treatments [14–16].

In the recent decade, there has been much progress in technologies in coronary computed tomography angiography (CCTA). This is able to provide more detailed information than CACS, including the degree of stenosis in individual coronary arteries, plaque characteristics, and the extent of any vascular remodeling [17–19]. We have reported that the evaluation of possible coronary artery disease (CAD) with multidetector CCTA is of importance in the early diagnosis of atherosclerosis in asymptomatic patients, particularly for assessing plaque characteristics [20].

Metabolic syndrome (MS) is a disorder, characterized by clusters of cardiovascular risk factors such as visceral obesity, high blood pressure, dyslipidemia, and impaired glucose tolerance [21]. Studies have confirmed that people with MS have an increased risk of cardiovascular morbidity and mortality [22].

There have been several studies supporting the relationship between MS and high burden of CACS [23, 24]. We have reported the association between MS and cardiovascular disease using multidetector CCTA [20]. However, longitudinal follow-up studies investigating the impact of MS on coronary vascular health using CACS or multidetector CCTA are limited [25]. Therefore, the aim of this study was to investigate the association of any progression of CACS and CAD with the severity of MS in a large Korean population, using CACS and CCTA.

## Methods

### Study population

This study was an observational, retrospective cohort study. Among people (*n* > 10,000 per year) who participated in a routine health check-up at Seoul National University Hospital Healthcare System Gangnam Center, a total of 17,390 subjects who had at least one or more cardiovascular risk factors, or who had atypical chest pain, underwent a CCTA from October 1, 2003 to December 31, 2012. Among them, 14,765 subjects who did not have follow-up CCTA scans until December 2013 were excluded. Next, 18 subjects who had medical histories of coronary revascularization at baseline were excluded. We also excluded those subjects whose CACS or coronary vascular health were not adequately evaluated (*n* = 97) or who had missing data for defining MS (*n* = 98).

**Fig. 1:**
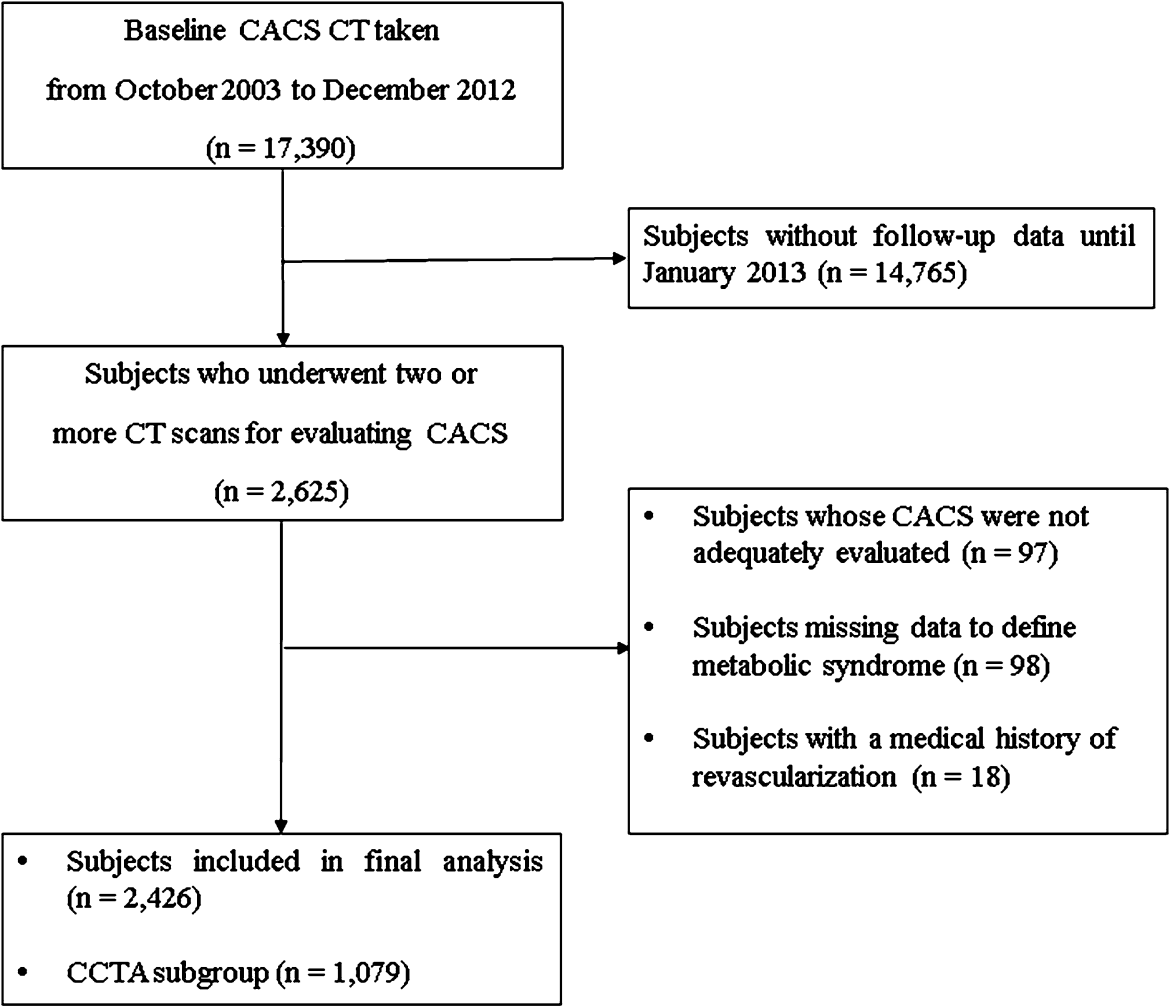
Selection of study participants

Finally, a total of 2426 subjects who had a baseline and a follow-up CACS at least 1 year apart were included in the analysis. The median follow-up period was 1197 days (interquartile range 766.0‒1687.5). Among these subjects, 1079 underwent CCTA tests at baseline and a follow-up CCTA test at least 1 year later. In this subgroup, we evaluated the progression of CAD by assessing the degree of coronary artery stenosis and presence of plaques and its characteristics. Selection of the study participants is outlined in Fig. 1.

The study protocol conformed to the ethical guidelines of the Declaration of Helsinki (2013). It was approved by the institutional review board of Seoul National University Hospital (IRB No. H-1501-003-635). All patients underwent multidetector CT after they had agreed to participate in the study and had provided informed consent after being informed of the possible risks of CT scanning.

### Anthropometric measurement and laboratory evaluations

On the day of the CT scan, anthropometric parameters such as height, body weight, waist circumference, and systolic and diastolic blood pressures were measured by trained nurses. Body mass index (BMI) was calculated as weight divided by height in kg/m^2^. Waist circumference was measured at the midpoint between the lower costal margin and the iliac crest.

Each subject completed a self-administered questionnaire, which included questions on demographic factors; a medical history covering diabetes mellitus (DM), dyslipidemia, hypertension, coronary revascularization, and drug use; family history of cardiovascular disease; and a social history including smoking habit, alcohol consumption, and levels of physical activity.

Smoking status was defined as follows: Patients were classified as current smokers if they currently smoked for at least 1 year. Patients were classified as nonsmokers if they had never smoked. Patients were classified as ex-smokers if they had smoked but quit. Patients were classified as nondrinkers or current drinkers by determining their average daily alcohol consumption. Nondrinkers were those who had not consumed alcohol within the past 30 days. Physical activity was classified into two categories: none or regular exercise. Regular exercise was defined as exercising more than three times a week (each session should be at least 30 min).

For biochemical tests, fasting plasma concentrations of glucose, total cholesterol, triglycerides, high-density lipoprotein (HDL)-cholesterol, and serum high-sensitivity C-reactive protein (hsCRP) were measured using an Architect Ci8200 analyzer (Abbott Laboratories, Abbott Park, IL, USA). Glycated hemoglobin (HbA1c) levels were measured using a COBAS INTEGRA 400 instrument (Roche Diagnostics GmbH, Mannheim, Germany). Plasma insulin concentrations were measured by radioimmunoassay (Linco, St. Louis, MO, USA). Aspartate/alanine aminotransferase (AST/ALT), gamma-glutamyl transpeptidase (γGT), and creatinine were measured using an Architect Ci8200 analyzer (Abbott Laboratories, Abbott Park, IL, USA). Low-density lipoprotein (LDL)-cholesterol concentration was calculated using the Friedewald equation [26]. In subjects with a triglyceride level ≥400 mg/dL, the measured LDL-cholesterol was used for analysis.

The homeostasis model assessment of insulin resistance (HOMA-IR) and pancreatic β-cell function (HOMA-β) were calculated as described by Matthews et al. [27]. HOMA-IR = fasting insulin (µIU/mL) × fasting plasma glucose (mg/dL)/405; HOMA-β = 360× fasting insulin (µIU/mL)/[fasting plasma glucose (mg/dL) − 63].

Diabetes mellitus was defined as a fasting plasma glucose level of 126 mg/dL (7 mmol/L) or higher or current antidiabetic treatment. Hypertension was defined as systolic/diastolic blood pressure measures greater than 140/90 mmHg or current treatment with antihypertensive medication. Dyslipidemia was defined as an LDL-cholesterol level of 160 mg/dL or higher or current use of a lipid-lowering agent.

### Evaluation of initial coronary CT angiography

In all subjects, coronary CT was performed either with a 16-slice scanner (Somatom Sensation 16; Siemens Medical Solutions, Forchheim, Germany) or with a 256-slice multidetector CT scanner (Brilliance iCT 256; Philips Medical Systems, Cleveland, OH, USA). A standard scanning protocol was applied, with 128 × 0.625 mm section collimation, 0.27 ms rotation time, 120 kV tube voltage, and 800 mA tube current. All scans were performed with electrocardiogram-gated dose modulation. Two experienced radiologists—blinded to all clinical information and patient demographics—analyzed all CT scans. The CACS was calculated quantitatively as described by Agatston et al. [28] using dedicated software (Rapidia 2.8; INFINITT, Seoul, South Korea).

Coronary artery stenosis and plaque characteristics were evaluated in the subjects who underwent multidetector CCTA. The coronary lumen cross-sectional diameter was traced at the maximal stenotic site and compared with the mean value for the proximal and distal reference sites. Coronary artery stenosis was evaluated according to five categories as follows: (1) normal or minimal (absence of plaque and no stenosis to plaque with <25 % stenosis); (2) mild (25–49 % stenosis); (3) moderate (50–69 % stenosis); (4) severe stenosis (70–99 % stenosis); and (5) occluded (100 % stenosis) [29, 30]. We recorded the degree of stenosis in each segment for one vessel and finally defined the maximal stenosis as being the grade of stenosis used in analysis for that vessel. Significant stenosis was defined as the presence of at least moderate stenosis. We also defined multivessel involvement when there was the presence of ≥50 % stenosis in at least two vessels.

Plaque characteristics were evaluated by arterial segment: left main artery; proximal, middle, and distal segment of left anterior descending artery; left circumferential artery; and right coronary artery. Each plaque was classified as calcified, mixed, or noncalcified type: (1) plaques that contained calcified tissue comprising ≥50 % of the plaque area were classified as calcified; (2) plaques with <50 % calcium in the plaque area were classified as mixed; and (3) plaques without any calcium were classified as noncalcified lesions [31]. If there was any plaque on a segment, we counted it as an atherosclerotic coronary segment. If there was a mixed or a noncalcified plaque, it was defined as a vulnerable atheromatous plaque [32, 33] (Additional file 1: Figure S1). Definition of metabolic syndrome

In accordance with modified National Cholesterol Education Program–Adult Treatment Panel III criteria [34, 35], an individual was classified as having MS if he or she had three or more of the flowing five criteria: (1) waist circumference ≥90 cm in men and ≥80 cm in women, using the International Obesity Task Force criteria for the Asian–Pacific population to determine waist circumference criteria [36]; (2) triglyceride levels ≥150 mg/dL (1.7 mmol/L); (3) HDL-cholesterol level <40 mg/dL (1.0 mmol/L) in men and <50 mg/dL (1.3 mmol/L) in women; (4) blood pressure ≥130/85 mmHg or the use of antihypertensive medication; and (5) fasting glucose level ≥100 mg/dL (6.1 mmol/L) or the self-reported use of antidiabetic medication (insulin or oral agents).

### Assessment of progression of coronary artery disease on cardiac CT

CACS progression was the main outcome measure of this study, which was assessed according to Berry et al. [37]. In patients with absence of any calcification at baseline (CACS = 0), progression was defined as CACS > 0 at follow-up. In subjects with a CACS > 0 and <100, progression was defined as an annualized increase of at least 10 Agatston units at follow-up, and for those with a CACS ≥ 100, an annualized percentage increase of ≥10 % at the follow-up was defined as progression.

For secondary outcome 1, progression of coronary artery stenosis was defined by the following conditions: (1) progression in the grade of stenosis at the vessel where the original stenosis was identified; and (2) generation of new stenosis at other segments. For secondary outcome 2, the development of new vulnerable plaque was defined as when any mixed or noncalcified plaque had developed in one or more segments.

### Statistical analysis

We compared data using Student’s *t* tests for continuous variables and χ^2^ tests for categorical variables. All data are expressed as the mean ± standard deviation (SD), median (interquartile range), or number (frequency). Log transformation was used for the statistical analysis of CACS, HOMA-IR, and hsCRP. χ^2^ tests with linear-by-linear associations were applied to examine the significance of any linear trend of CAD progression according to the number of MS components. A Cox proportional hazard model was used to investigate any independent effect of MS on the progression of CAD. The analyses were performed using IBM SPSS Statistics for Windows (version 20.0; IBM Corp., Armonk, NY, USA). Data with *P* < 0.05 were considered significant.

## Results

### Baseline clinical characteristics

Baseline characteristics and biochemical parameters were compared according to the presence or absence of MS (Table 1). Baseline characteristics according to the gender were presented in the Additional file 1: Table S1. At baseline, 825 subjects had MS (34.0 % of total population). Their mean age was about 56 years and similar between the MS group and those without MS. The subjects with MS had higher BMI, larger waist circumference, and higher blood pressure, as expected with the definition of MS.

**Table 1:** Baseline clinical and biochemical characteristics in subjects with and without metabolic syndrome (MS)

	MS (*n* = 825)	Non-MS (*n* = 1601)	*P*
Clinical and anthropometric parameters			
Age, years	56.6 (7.5)	56.2 ± 7.2	0.180
No. (%) of male	661 (80.0)	1278 (79.8)	0.873
Height, cm	167.5 (7.6)	166.7 (7.4)	0.010
Weight, kg	73.8 (10.3)	66.5 (9.1)	<0.001
BMI, kg/m^2^	26.2 (2.6)	23.9 (2.3)	<0.001
Waist circumference, cm	92.7 (6.6)	85.9 (6.4)	<0.001
SBP, mmHg	127.5 (13.8)	118.5 (14.3)	<0.001
DBP, mmHg	83.8 (10.5)	77.8 (10.5)	<0.001
Biochemical parameters			
Fasting glucose, mmol/L	6.37 (1.30)	5.49 (0.93)	<0.001
HbA1c, %	6.1 (0.8)	5.8 (0.6)	<0.001
Insulin, μU/mL	12.3 (5.6)	8.5 (4.1)	<0.001
HOMA-IR^a^	3.48 (1.74)	2.12 (1.10)	<0.001
HOMA-β^a^	97.6 (57.8)	95.0 (54.2)	0.434
AST, IU/L	28.9 (14.0)	24.8 (9.3)	<0.001
ALT, IU/L	34.3 (21.5)	26.2 (14.7)	<0.001
γGT, IU/L	55.6 (59.5)	35.7 (30.8)	<0.001
Creatinine, µmol/L	93.70 (17.68)	93.70 (15.03)	0.884
Total cholesterol, mmol/L	5.13 (0.93)	5.18 (0.88)	0.147
Triglyceride, mmol/L	2.04 (104.1)	1.18 (0.58)	<0.001
HDL-cholesterol, mmol/L	1.21 (0.29)	1.42 (0.32)	<0.001
LDL-cholesterol, mmol/L	3.02 (0.87)	3.23 (0.81)	<0.001
hsCRP^a^, mg/dL	0.17 (0.42)	0.14 (0.44)	0.118
Comorbidity and lifestyles			
Hypertension (%)	70.9	32.8	<0.001
Diabetes mellitus (%)	32.3	12.7	<0.001
Dyslipidemia (%)	37.5	27.6	<0.001
Medication			
Hypertension (%)	58.0	23.5	<0.001
Diabetes mellitus (%)	18.9	7.5	<0.001
Dyslipidemia (%)	36.0	22.8	<0.001
Smoking status			0.119
Current smoker (%)	12.7	11.6	
Ex-smoker (%)	43.2	39.9	
Never smoker (%)	44.1	48.5	
Current drinker (%)	74.3	73.0	0.673
Regular exercise (%)	34.5	35.5	0.654

Data are shown as the mean and (SD) or percentages

*MS* metabolic syndrome, *SBP* systolic blood pressure, *DBP* diastolic blood pressure, *HbA1c* glycated hemoglobin, *HOMA-IR* homeostasis model assessment of insulin resistance, *HOMA-β* homeostasis model assessment of pancreatic β-cell function, *γGT* γ-glutamyl transpeptidase, *HDL* high-density lipoprotein, *LDL* low-density lipoprotein, *hsCRP* high sensitivity C-reactive protein

^a^Log-transformed values were used for analysis

The MS group had a significantly greater impairment in glucose tolerance and higher insulin resistance than did the group without MS (*P* < 0.05). The HOMA-β results were not different between the two groups. Liver enzyme activities such as the levels of AST, ALT, and γGT were significantly higher in the MS group than in the group without it (*P* < 0.05). Serum triglyceride levels were higher and HDL-cholesterol levels were significantly lower in the MS group than in the groups without MS (both *P* < 0.05). Serum LDL-cholesterol levels were significantly higher in the group without MS, but there lower LDL-cholesterol level in the MS group could be attributed to their used of medication. The rates of hypertension, DM, and dyslipidemia were significantly higher in the MS group than in the group without MS. The proportions of subjects who were taking medications for hypertension, DM, and dyslipidemia were also significantly greater in the MS group than in the group without MS. Smoking status, alcohol consumption, and exercise habits did not differ between the two groups.

### Cardiac CT findings according to the presence of metabolic syndrome

At baseline, the mean ± SD of CACS in the MS group was significantly greater than that in the group without MS (103.7 ± 270.9 vs. 71.3 ± 225.1, *P* < 0.01). The proportion of subjects with coronary calcium (CACS > 0) was also higher in the MS group than in the group without MS (54.4 vs. 45.8 %; *P* < 0.001).

In the MS group, 52.6 % of subjects showed CACS progression, while in the group without MS, 40.4 % of subjects had progressed (*P* < 0.001). In the subgroup of 1079 subjects with data concerning the degree of coronary artery stenosis and coronary plaque using CCTA at baseline, significantly more subjects with MS had significant stenosis (13.6 vs. 8.9 %; *P* = 0.017), multivessel involvement (4.2 vs. 1.7 %; *P* = 0.015), and coronary plaques (55.1 vs. 43.3 %; *P* < 0.001) than did those without MS. In the follow-up, the subjects with MS showed a trend of progression of stenosis than did the group without MS (28.6 vs. 23.4 %; *P* = 0.067). Furthermore, the development of vulnerable plaque (mixed or noncalcified types) was larger in the MS group than group without MS with borderline significance (Table 2).

**Table 2:** Comparison of cardiac computed tomography findings between subjects with and without metabolic syndrome (MS)

	MS	Non-MS	*P*
Baseline data			
CACS	*n* = 825	*n* = 1601	
Initial CACS, median (IQR)	11.1 (0‒98.5)	0.0 (0‒43.0)	<0.001^a^
Prevalence of coronary calcification, n (%)	449 (54.4)	734 (45.8)	<0.001
CCTA	*n* = 381	*n* = 698	
Significant stenosis, n (%)	52 (13.6)	62 (8.9)	0.017
Multivessel disease, n (%)	16 (4.2)	12 (1.7)	0.025
Any plaque, n (%)	210 (55.1)	302 (43.3)	<0.001
Plaque type			
Calcified, n (%)	156 (40.9)	233 (33.4)	0.463
Mixed, n (%)	60 (15.7)	82 (11.7)	0.764
Non-calcified, n (%)	41 (10.8)	63 (9.0)	0.739
Follow up data			
CACS	*n* = 825	*n* = 1601	
FU interval, median (IQR), days	1285 (763.3‒1640.3)	1292 (763.5‒1703.0)	0.808
Follow-up CACS, median (IQR)	45.3 (0‒225.3)	10.4 (0‒115.4)	<0.001^a^
Progression of CACS, n (%)	434 (52.6)	647 (40.4)	<0.001
CCTA	*n* = 381	*n* = 698	
FU interval, median (IQR), days	1106 (735.0‒1484.6)	1102 (741.8‒1465.3)	0.948
Progression of stenosis, n (%)	109 (28.6)	163 (23.4)	0.067
Development of vulnerable plaque, n (%)	95 (24.9)	138 (19.8)	0.053

*CCTA* coronary computed tomography angiography, *CACS* coronary artery calcium score, *FU* follow-up, *IQR* interquartile range

^a^Log-transformed values were used for analysis

**Fig. 2:**
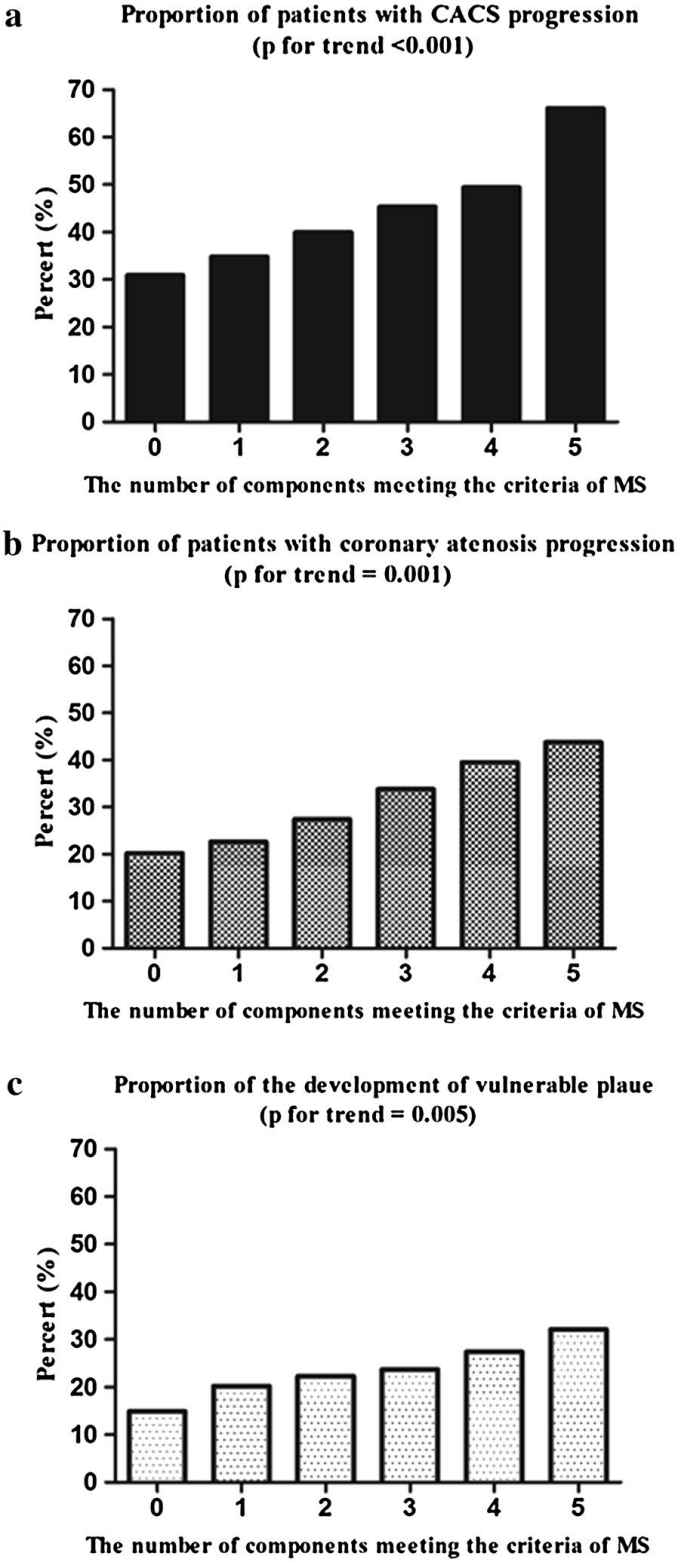
Proportions of CAD progression according to the number of the metabolic syndrome (MS) components present. **a** Proportion of patients with CACS progression. **b** Proportions of progression evaluated by the degree of coronary stenosis. **c** Proportions of the development of vulnerable plaque, according to the numbers of components of MS

Next, we evaluated any association between the number of MS components and progression of CAD. As the number of MS components increased, so did the progression of CACS. The proportion of subjects with CACS progression increased gradually from 30.9 % in subjects without any MS component to 66.0 % in those with all five components (*P* for trend <0.001; Fig. 2a). A similar trend was observed when the progression was evaluated using the degree of coronary artery stenosis (*P* for trend = 0.001; Fig. 2b) or the development of vulnerable plaque (*P* for trend = 0.005; Fig. 2c).

### Multivariable analysis of primary and secondary outcomes

We performed multivariable analyses with Cox proportional hazard models to investigate any independent effect of MS on the progression of CAD (Table 3). First, the presence of MS was significantly associated with the progression of CACS after adjusting for age and sex (hazard ratio [HR], 1.68, 95 % confidence interval [CI], 1.39–2.03, *P* < 0.001) (**Model A**) and additional adjusting for smoking habit and family history of CAD (HR 1.67; 95 % CI, 1.38–2.20: *P* < 0.001) (**Model B**). After further adjusting for BMI, and the levels of LDL-cholesterol and HbA1c, the presence of MS was significantly linked with the progression of CACS with slight attenuation (HR 1.32; 95 % CI 1.06–1.64; *P* = 0.012) (**Model C**).

**Table 3:** Association of metabolic syndrome with progression of CAD after multivariable adjustment

	Progression of CACS			Progression of coronary artery stenosis or development of vulnerable plaque^a^		
	HR	95 % CI	*P*	HR	95 % CI	*P*
Model A						
Adjusted for age and sex	1.68	1.39–2.03	<0.001	1.45	1.06–1.99	0.021
Model B						
Adjusted for age, sex, smoking, and family history of CAD	1.67	1.38–2.20	<0.001	1.44	1.05–1.99	0.025
Model C						
Adjusted for age, sex, smoking, family history of CAD, BMI, LDL-cholesterol, and HbA1c	1.32	1.06–1.64	0.012	1.47	1.01–2.15	0.045

*BMI* body mass index, *CAD* coronary artery disease, *CACS* coronary artery calcium score, *HbA1c* glycated hemoglobin, *LDL* low-density lipoprotein

^a^Vulnerable plaques contain mixed and noncalcified plaques

Next, we performed the same analysis using CCTA data. The presence of MS was significantly associated with the progression of coronary artery stenosis or the development of vulnerable plaque after adjusting for age and sex (HR 1.45; 95 % CI 1.06–1.99; *P* = 0.021) (**Model A**) and additional adjusting for smoking habit and a family history of CAD (HR 1.44; 95 % CI 1.05–1.99; *P* = 0.025) (**Model B**). After further adjusting for BMI, and the levels of LDL-cholesterol and HbA1c, a significant association of the presence of MS with the progression of coronary artery stenosis or development of vulnerable plaque was maintained (HR 1.47; 95 % CI 1.13–2.15; *P* = 0.045) (**Model C**).

## Discussion

In this large retrospective longitudinal study, the subjects with MS had more CAD than those without MS at baseline. More importantly, those with MS showed rapid development or progression of CACS, coronary artery stenosis, and vulnerable plaque in the longitudinal follow-up, with a positive association with the number of MS components. MS was found to be an independent predictor for CAD progression even after adjusting for multiple relevant risk factors.

The CACS, one of the indicators of subclinical CAD, is correlated strongly with the extent of atherosclerosis [9]. According to the Jackson Heart Study, the presence of a high CACS was directly associated with the incidence of cardiovascular disease [38]. High CACS was associated with arterial stiffness measured by aortic pulse wave velocity, which is another potential predictor of cardiovascular events [39]. There have been many cross-sectional studies investigating the association between MS and the high burden of coronary artery calcium [40–44]. Wong et al. [45] showed that individuals with MS had a greater degree of progression of CACS compared with those without MS. Moreover, it was reported that nonalcoholic fatty liver disease, which is now recognized as the hepatic manifestation of MS and insulin resistance, has a significant association with CACS [46, 47]. These studies suggest that CACS is an effective marker for evaluating CAD and further forms of CVD, as mentioned above.

However, measurement of calcium deposition only in coronary arteries seems not sufficient to evaluate CAD precisely. Even though CACS is an established surrogate marker for coronary atherosclerosis [13], it cannot provide detailed information on CAD such as the degree of coronary artery stenosis or plaque characteristics. Direct assessment of individual coronary vessels seems to be more helpful for assessing coronary vascular health than simple calcium deposition. Detailed evaluation of plaque characteristics is also of importance in this context. In fact, mixed or noncalcified plaques occur more frequently in patients with an acute coronary syndrome than in those with stable angina [48], and are known to be associated with a higher all-cause mortality outcome than calcified plaques [49]. These findings suggest that vulnerable plaques might trigger plaque rupture and are more associated with poor cardiovascular outcome [32].

So far, only a few studies have shown an association between MS and the degree of stenosis/plaque characteristics assessed by CCTA [20, 50]. We have reported previously that MS was significantly associated with noncalcified/mixed plaques in a large number of study participants, suggesting that CCTA is a useful tool in the detailed evaluation of subclinical coronary atherosclerosis for individuals at high risk [20]. Furthermore, few studies have evaluated the influence of MS on the development or progression of CAD using CCTA in longitudinal studies [25]. To the best of our knowledge, our study is the largest that has investigated the influence of MS on development and/or progression of CAD as assessed comprehensively by CCTA in a longitudinal setting.

Here, we confirmed previous reports by demonstrating that people with MS had a greater extent of coronary calcification and a higher prevalence of significant coronary stenosis, multivessel involvement, and coronary plaques than those without MS [40–43]. Furthermore, we found a significant rate of progression of CACS in the follow-up. Significantly more progression of coronary artery stenosis or greater development of vulnerable plaque was also found in the subjects with MS than those without MS in the follow-up with CCTA. Among all the subjects (*n* = 2426) enrolled in this study, 1079 underwent CCTA tests both at baseline and at least 1 year later. Of these subjects, 155 (14.4 %) had significant progression of coronary artery stenosis and/or development of vulnerable plaques without CACS progression. This finding supports our conclusion that CCTA is able to provide more sensitive information about CAD progression than simple CACS.

This study had several strengths. First, comprehensive evaluations including medical history, anthropometric measurements, and biochemical parameters were possible because data were collected through the general health check-up. Second, we analyzed long-term follow-up data, so that we could determine any independent association of MS with the progression of CAD. Third, we analyzed detailed information about the degree of coronary stenosis and plaque characteristics using CCTA with advanced technology.

Our study also had several limitations. First, it was a retrospective, longitudinal study so there might have been confounders that were not corrected sufficiently. Second, the study participants, who voluntarily underwent a health check-up, might not have been representative of the general Korean population. Also, their health-seeking behavior and the interventions possibly taken after the baseline evaluation might have changed their cardiovascular risk profiles during the study period and could attenuate the relationship between MS and the progression of CAD. Third, a comprehensive evaluation of CAD using CCTA was available in only 44.5 % of the study participants.

## Conclusions

We confirmed that MS is associated with coronary atherosclerosis as evidenced by significant coronary artery stenosis, multivessel involvement, and/or high plaque burden as well as high CACS. Subjects with MS showed greater progression not only in the CACS, but also in the degree of coronary stenosis and vulnerable plaque formation. Thus, our study has confirmed that MS is an independent risk factor for the progression of CAD even after adjusting for known cardiovascular risk factors. These findings suggest that more vigilant screening for subclinical CAD is helpful in individual with MS and more aggressive management for cardiovascular risk factors is required to prevent any progression of CAD in these subjects.

## Additional file

10.1186/s12933-016-0404-7 Comparison of baseline clinical and biochemical characteristics by genders. **Figure S1.** Progression of coronary artery disease from baseline cardiac computed tomography scans to 3–4 years follow-up scans. 1) Increase of coronary artery calcium deposition (**A** and **B**), 2) progression of coronary artery stenosis (**C** and **D**), 3) development of noncalcified plaque (**E** and **F**) 3–4 years.
