# Supplementary material for: Impact of metabolic syndrome on the progression of coronary calcium and of coronary artery disease assessed by repeated cardiac computed tomography scans
Source: Cardiovasc Diabetol. 2016 Jun 28;15:92. doi: 10.1186/s12933-016-0404-7 (PMC4924293; doi:10.1186/s12933-016-0404-7)
Supplement: Supplementary file 1 — 10.1186/s12933-016-0404-7 Comparison of baseline clinical and biochemical characteristics by genders. Figure S1. Progression of coronary artery disease from baseline cardiac computed tomography scans to 3–4 years follow-up scans. 1) Increase of coronary artery calcium deposition (A and B), 2) progression of coronary artery stenosis (C and D), 3) development of noncalcified plaque (E and F) 3–4 years. [file 12933_2016_404_MOESM1_ESM.docx]

**Figure S1. Progression of coronary artery disease from baseline cardiac computed tomography scans to 3-4 years follow-up scans.** 1) Increase of coronary artery calcium deposition (**A and B**), 2) Progression of coronary artery stenosis (**C and D**), 3) Development of noncalcified plaque (**E and F**). 3-4 years


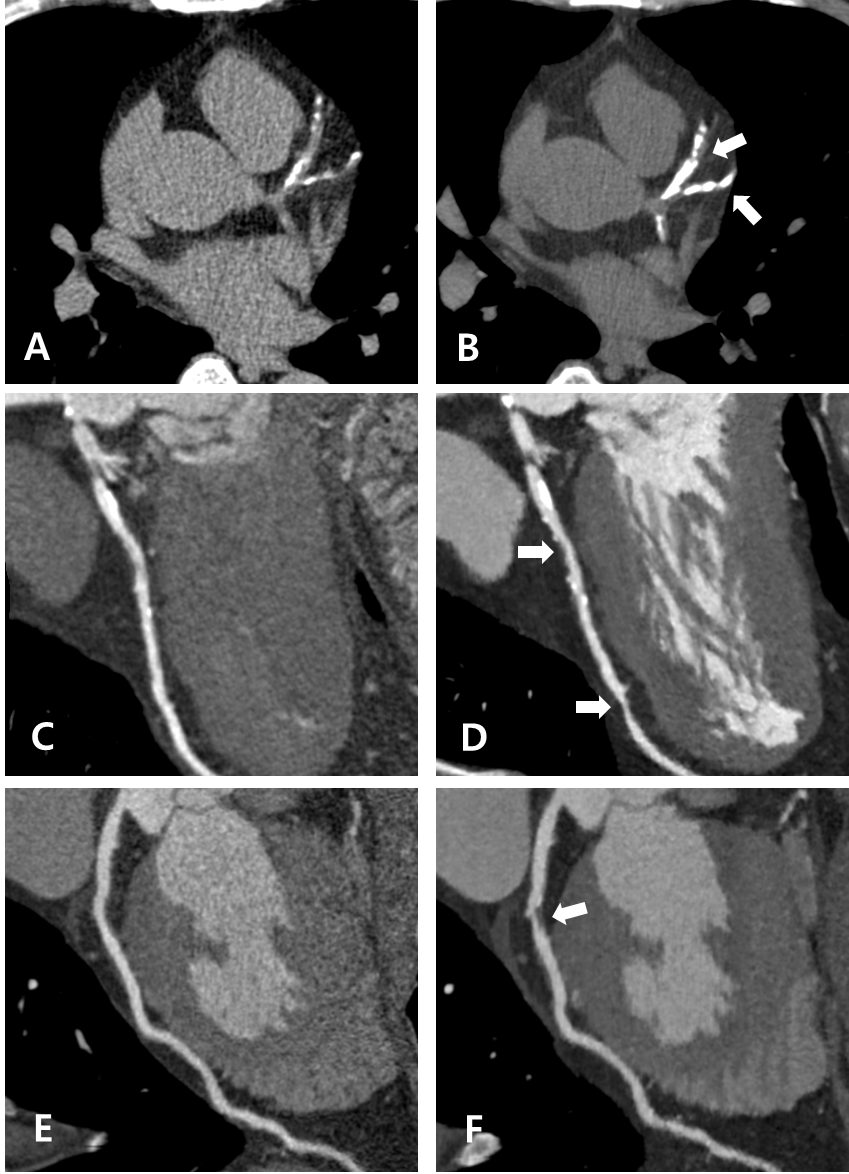


| **Table S1. Comparison of Baseline Clinical and biochemical characteristics by genders** | | | |
| --- | --- | --- | --- |
|  | MEN (n = 1939) | WOMEN (n=487) | p Value |
| Age, mean (SD), years | 56.1 (7.3) | 57.6 (7.1) | <0.001 |
| Height (cm) | 169.5 (5.6) | 156.8 (5.1) | <0.001 |
| Weight (kg) | 71.8 (8.5) | 57.6 (7.8) | <0.001 |
| BMI (kg/m^2^) | 25.0 (2.5) | 23.4 (2.9) | <0.001 |
| Waist circumference, mean (SD), cm | 89.0 (6.7) | 84.8 (8.3) | <0.001 |
| SBP, mean (SD), mm Hg | 121.8 (14.2) | 120.4 (16.9) | 0.061 |
| DBP, mean (SD), mm Hg | 81.0 (10.4) | 75.2 (11.4) | <0.001 |
| ***Biochemical parameters*** |  |  |  |
| Fasting glucose, mean (SD), mmol/L | 5.85 (1.13) | 5.55 (1.16) | <0.001 |
| HbA1c, mean (SD), % | 5.9 (0.7) | 5.9 (0.6) | 0.878 |
| Insulin, mean (SD), μU/mL | 10.0 (5.0) | 9.3 (5.0) | 0.057 |
| HOMA-IR, mean (SD) | 2.67 (1.52) | 2.33 (1.43) | 0.001 |
| HOMA-B, mean (SD) | 93.8 (54.4) | 104.9 (59.3) | 0.007 |
| AST mean (SD), IU/L | 26.7 (11.8) | 24.5 (8.9) | <0.001 |
| ALT mean (SD), IU/L | 30.2 (18.1) | 23.8 (15.2) | <0.001 |
| γGT mean (SD), IU/L | 46.8 (25.2) | 45.3 (31..6) | <0.001 |
| Creatinine mean (SD), µmol/L | 84.64 (11.44) | 67.10 (13.72) | 0.884 |
| Total cholesterol mean (SD), mmol/L | 5.11 (0.88) | 5.38 (0.89) | 0.147 |
| Triglyceride, mean (SD), mmol/L | 1.54 (0.97) | 1.18 (0.61) | <0.001 |
| HDL-cholesterol, mean (SD), mmol/L | 1.31 (0.30) | 1.51 (0.36) | <0.001 |
| LDL-cholesterol, mean (SD), mmol/L | 3.12 (0.83) | 3.33 (0.83) | <0.001 |
| hs-CRP, mean (SD), mg/dL | 0.16 (0.46) | 0.13 (0.32) | 0.287 |
| ***Comorbidity and lifestyles*** |  |  |  |
| Hypertension, n (%) | 671 (47.1) | 143 (42.7) | 0.161 |
| Diabetes Mellitus, n (%) | 292 (20.5) | 51 (15.4) | 0.038 |
| Dyslipidemia, n (%) | 580 (29.9) | 171 (35.1) | 0.028 |
| Metabolic syndrome, n (%) | 661 (34.1) | 164 (33.7) | 0.873 |
| Medication |  |  |  |
| Hypertension, n (%) | 510 (35.8) | 118 (35.2) | 0.899 |
| Diabetes mellitus, n (%) | 177 (12.4) | 26 (7.9) | 0.022 |
| Dyslipidemia, n (%) | 388 (26.4) | 111 (33.4) | 0.012 |
| Smoking status (%) |  |  | <0.001 |
| Current smoker | 287 (14.8) | 4 (0.8) |  |
| Ex-smoker, n (%) | 984 (50.7) | 10 (2.1) |  |
| Never smoker, n (%) | 668 (34.5) | 473 (97.1) |  |
| Current drinker, n (%) | 83.1 | 22.8 | <0.001 |
| Regular exercise, n (%) | 37.8 | 25.1 | <0.001 |
| Abbreviations: MS, metabolic syndrome; SBP, systolic blood pressure; DBP, diastolic blood pressure; HbA1c, hemoglobin A1c; HOMA-IR, homeostasis model assessment - insulin resistance; HOMA-B, homeostasis model assessment - beta cell function; HDL, high-density lipoprotein; LDL, low-density lipoprotein; CRP, c-reactive protein | | | |

.
